# Learning Local–Global Multiple Correlation Filters for Robust Visual Tracking with Kalman Filter Redetection

**DOI:** 10.3390/s21041129

**Published:** 2021-02-05

**Authors:** Jianming Zhang, Yang Liu, Hehua Liu, Jin Wang

**Affiliations:** Hunan Provincial Key Laboratory of Intelligent Processing of Big Data on Transportation, School of Computer and Communication Engineering, Changsha University of Science and Technology, Changsha 410114, China; jmzhang@csust.edu.cn (J.Z.); yang_liu@stu.csust.edu.cn (Y.L.); lhh@stu.csust.edu.cn (H.L.)

**Keywords:** object tracking, correlation filter, convolutional neural networks, local–global collaborative strategy, Kalman filter

## Abstract

Visual object tracking is a significant technology for camera-based sensor networks applications. Multilayer convolutional features comprehensively used in correlation filter (CF)-based tracking algorithms have achieved excellent performance. However, there are tracking failures in some challenging situations because ordinary features are not able to well represent the object appearance variations and the correlation filters are updated irrationally. In this paper, we propose a local–global multiple correlation filters (LGCF) tracking algorithm for edge computing systems capturing moving targets, such as vehicles and pedestrians. First, we construct a global correlation filter model with deep convolutional features, and choose horizontal or vertical division according to the aspect ratio to build two local filters with hand-crafted features. Then, we propose a local–global collaborative strategy to exchange information between local and global correlation filters. This strategy can avoid the wrong learning of the object appearance model. Finally, we propose a time-space peak to sidelobe ratio (TSPSR) to evaluate the stability of the current CF. When the estimated results of the current CF are not reliable, the Kalman filter redetection (KFR) model would be enabled to recapture the object. The experimental results show that our presented algorithm achieves better performances on OTB-2013 and OTB-2015 compared with the other latest 12 tracking algorithms. Moreover, our algorithm handles various challenges in object tracking well.

## 1. Introduction

With the development of artificial intelligence, sensor networks nodes equipped with more sophisticated sensing units such as cameras have become ubiquitous in cities. Camera-based sensor networks have been widely used in intelligent transportation and smart cities. Because big video data are generated by cameras, the video analysis of smart nodes or edge servers needs to be energy efficient and intelligent. Visual object tracking is one of the fundamental problems in various application fields of computer vision, such as autonomous driving, precise navigation, video surveillance, and human–computer interaction. However, because of partial or complete occlusion, fast motion, lighting changes, scale changes, and similar objects and complex backgrounds, high-precision and robust visual target tracking is still a difficult task.

Building a target appearance model with strong representation capability is one of the keys to achieving the high precision and robustness of object tracking. According to the different categories of target appearance representation models used, the methods of visual object tracking can be divided into two types: generative methods [1,2] and discriminative methods [3,4]. The generative methods extract the target features for learning the appearance model representing the target at first, and then search the image area for pattern matching. The discriminative methods use the positive and negative samples collected from each frame to train a binary classifier that distinguishes the target from the background; the candidate sample with the highest confidence in the current frame being selected as the tracking result. In recent years, discriminative correlation filter (DCF)-based trackers [5,6,7] have gained more and more attention from researchers due to their excellent performance. The DCF-based trackers can convert all samples into a diagonal matrix and transform the calculation into the frequency domain by Fourier transform, which greatly improves the speed of obtaining the solution to the correlation filter. The extraction method of the target appearance features mostly determines the performance of the DCF-based tracking algorithm. In order to train high-quality CF, it is necessary for the tracking algorithm to select an appropriate descriptor or a combination of multiple feature descriptors including a histogram of oriented gradient features (HoG) [8], color names (CN) features [9], Point of Interest features [10], Haar-like rectangular features [11], superpixel features [12], etc. This work uses a local–global multiple correlation filters model constructed by convolutional features and hand-crafted features to improve tracking performance.

### 1.1. Motivation

As a result of the strong discriminative capabilities of the convolutional features extracted by deep convolutional neural networks (CNN), they have been widely used in correlation filter tracking algorithms [13,14,15,16]. The features in the earlier layers of CNN retain high spatial resolution information that can be used for precise localization for targets, while rich semantic information contained in the features in the higher layers can be used to deal with the dramatic appearance changes for the target. General combination of low-level and high-level features can greatly improve the performance of the tracker, but there are still some limitations.

**Figure 1:**
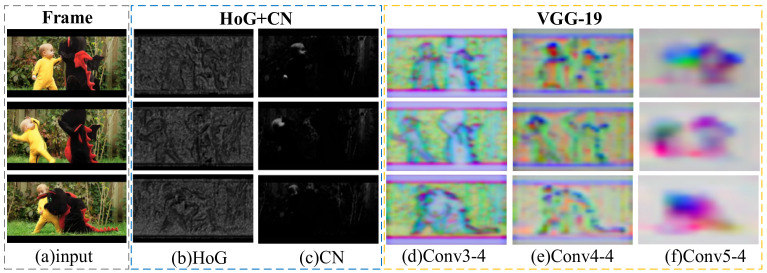
Visualization of multi-level features. (**a**) Three challenging frames on the DragonBaby sequence. (**b**) Histogram of oriented gradient (HoG). (**c**) Color Name. (**d**–**f**) are high-level features extracted from Conv3-4, Conv4-4, and Conv5-4 of VGGNet-19.

Deep features are used to represent targets in the CF-based trackers [17,18,19,20]. Compared with deep features, hand-crafted features are more robust to occlusion and shape changes. We visualize the above features as shown in Figure 1. It is important to improve the accuracy and robustness of the tracker to find a reasonable combination of multiple features. Moreover, due to the interference of factors such as occlusions and illumination changes during the tracking process, errors will gradually accumulate through the online update of CF, which will cause model drift and loss. Trackers with a single model can only learn one kind of appearance model, therefore the targets are prone to be lost. One recent approach [21] proposed a part-based multiple correlation filter tracking method that relies on the cooperation between a global filter and several part filters. However, this method uses only hand-crafted features, which makes it difficult to achieve excellent results. Thus, it is imperative to construct a reasonable multiple correlation filters cooperation mechanism.

These CF-based trackers have achieved rather good performance in visual object tracking. However, because they can only use the maximum value of the response map as the predicted result for candidate target position, it may be unreliable when the target is out of view or completely occluded. More specifically, the response map may have multiple peaks or a decrease in maximum value when it comes to target occlusion or out of view objects. Therefore, an effective redetection mechanism is especially important in the tracking algorithm [22,23,24], when target occlusion and out of view occurs.

### 1.2. Contribution

In view of the above-mentioned two points, we put forward a local–global multiple correlation filter model and Kalman filter redetection mechanism to achieve accurate and robust object tracking. The main contributions are outlined below:

1. We propose an intelligent local–global multiple correlation filter (LGCF) learning model. Our global filter uses multilayer CNN features to capture the whole appearance of the target. At the same time, the object is divided into two equal sized parts to construct local filters, which use handcraft features to capture the partial appearance of the target. We choose a position estimation method from coarse to fine and use a combination of multiple features to achieve accurate object tracking.
2. We introduce an effective method where the target can be tracked accurately through Kalman filter redetection (KFR). We also propose time-space peak to sidelobe ratio (TSPSR) as a confidence value to measure the reliability of the current estimated position. Compared with CF with a different appearance model, we comprehensively use the time and motion information of the target instead of just the appearance information. If the tracking result of the correlation filter with the appearance model is unreliable, this method can make the target position regained to continue the tracking procedure.
3. Based on the local–global learning model, we propose a collaborative update strategy with multiple correlation filters. In the update phase, we divide the global filter into three cases according to the state of the local filter: normal update, slow update, and no update. This method can effectively prevent the collaborative filters from learning the wrong target appearance.
4. We have evaluated the performance of the proposed LGCF algorithm on the OTB-2013 [25] and OTB-2015 [26] datasets, and verified the effectiveness of each contribution. Experimental data show that this method can improve the tracking accuracy and success rate effectively. The precision and success rates on the OTB-2013 reached 90.6% and 64.9%, respectively. On OTB-2015, the accuracy of our tracker reached 86.2%, and the success rate reached 61.6%.

The remainder of this paper is organized as follows: Section 2 briefly introduces related work. The proposed tracking method is described in detail in Section 3. Experimental results are reported and analyzed in Section 4. We conclude our paper in Section 5.

## 2. Related Work

In this section, three types of tracking algorithms related to our algorithm are mainly introduced: tracking by correlation filters, tracking by deep learning, and tracking by Kalman filter (KF).

### 2.1. Tracking by CF

A tracking algorithm based on a correlation filter transforms the process of solving the filter into the frequency domain through a fast Fourier transform (FFT) to accelerate the calculation. Bolme et al. proposed the Minimum Output Sum of Squared Errors (MOSSE) [5] algorithm in 2010, which first introduced CF into visual tracking. The Kernelized Correlation Filter Tracker (KCF) uses CN and HoG features to compose multi-channel features, and uses kernels to map the calculation of low-dimensional feature space to linearly separable high-dimensional feature space [7]. Henriques et al. [8] propose Kernel Regularized Least Squares, which improves the speed of classifying samples in high-dimensional feature space. Bertinetto et al. [27] use statistical color histogram and HoG features to represent the target to train the CF. The fusion of these two features further improves tracking accuracy. With the widespread application of convolutional neural networks [28,29], the hierarchical convolutional features tracker (HCF) [17] and Correlation Filters with Weighted Convolution Responses (CFWCR) [18] choose deep features extracted from VGGNet to train CF, and the tracking performance is further improved. Danelljan et al. [30] establish an interpolation model for training samples in [31], combine features in different resolutions to obtain feature maps with continuous spatial resolution, and use Gaussian mixture models to classify samples in the training set so that the similar samples are in one group. This method avoids overfitting between the samples in consequent frames. References [32,33] introduce multiscale estimation models. These two models have become commonly used in the process of object tracking. In order to solve the boundary effect caused by the periodic hypothesis, a spatial regularization term is introduced [6]. In the block object tracking [34], a probabilistic model is proposed to estimate the distribution of reliable patches under the sequential Monte Carlo framework. However, it is difficult for a filter trained by one type of feature to deal with the effects of multiple factors such as illumination and deformation at the same time. Therefore, we must find a reasonable way to combine multiple features. In our work, we comprehensively consider the invariance of hand-crafted features to geometry and illumination, as well as the fine-grained and semantic information of convolutional features. Therefore, the global filters are learned by multilayer convolutional features, while local filters are learned by the combination of HoG and CN features.

### 2.2. Tracking by Deep Learning

Deep convolutional neural networks require large-scale data for training, but this does not stop them from being widely used in object tracking due to their strong feature characterization capabilities. Some trackers directly apply deep features to the DCF framework, such as the Hedged deep tracker (HDT) [15]. Learning continuous convolution operators (CCOT) [31] and efficient convolution operators (ECO) [30] integrate the deep feature maps in different resolutions after interpolation. In addition, the Fully-convolutional Siamese network (SiamFC) [35] uses an end-to-end learning method and deep features to find a similarity between the target and the templates for object tracking. Dynamic Siamese (Dsiam) [36] can learn the appearance changes of the target online and use consequent video sequences for training to improve the tracking performance. The Siamese region proposal network (SiamRPN) [37] considers the tracking problem as a detection problem by utilizing a Regional Proposal Network (RPN) commonly used in object detection to obtain more accurate predictions of the tracking targets. A combined online tracking and segmentation method named SiamMask [38] introduces a binary mask branch to improve speed and accuracy in object tracking. Despite great success, trackers based on deep learning are still limited in many ways. On the one hand, a CNN model trained by a generic dataset such as ImageNet [39] is suitable for unspecified targets and is easily affected by background clutter. On the other hand, a tracker based on deep learning usually only learns the positive samples around the target and executes the strategy of not updating or slowly updating the model during the tracking process, which are easy to be influenced by occlusion or out of view. In this paper, we use multi-layer deep convolutional features extracted from VGGNet-19 [40] to train a global filter in the way of weighted combination. The global filter is used to achieve the coarse localization of the target. Then, HoG and CN features combined into 41-dimensional features are utilized to train our local filters. The local filters are used to achieve fine target localization and correct the coarse position estimation of global filters.

### 2.3. Tracking by KF

Kalman filter is an optimal linear recursive filter algorithm that can be easily implemented in a computer. It can effectively deal with problems relevant to filters under linear Gaussian. Multi-hierarchical Independent Correlation Filters for Visual Tracking (MFT) [41] uses KF to construct a motion estimation module to predict the trajectory of the tracking target. Reference [42] proposes Strong Tracking Marginalized Kalman Filter (STMKF) for robust tracking with an edge Kalman filter. By using an attenuation factor in the Marginalized Kalman Filter (MKF), this reduces the impact of the previous filter on the current filter, considering the object in 3D space. Reference [43] analyzes the performance of extended KF using the azimuth to track single and multiple objects, respectively. Reference [44] compares the performance of the Kalman filter and particle filter for two-dimensional visual multi-object tracking in different environments. Reference [45] describes a method for detection and tracking on moving objects using KF. Aimed at cases where the object is occluded, this method can only rely on the state of the target in the previous frame to predict the target position in the current frame. However, the accuracy of the KF-based tracker is limited, and the mechanism of KF that needs to update every frame is likely to cause an accumulation of errors. Our method is similar to that in [45]. We use the tracking results of CF to replace target detection results. When the tracking target is occluded, the tracking result of the CF will drift or be lost. At this time, our KFR module will predict the target position in the current frame according to the status information of the target in the previous frame.

## 3. Our Approach

### 3.1. Overview

**Figure 2:**
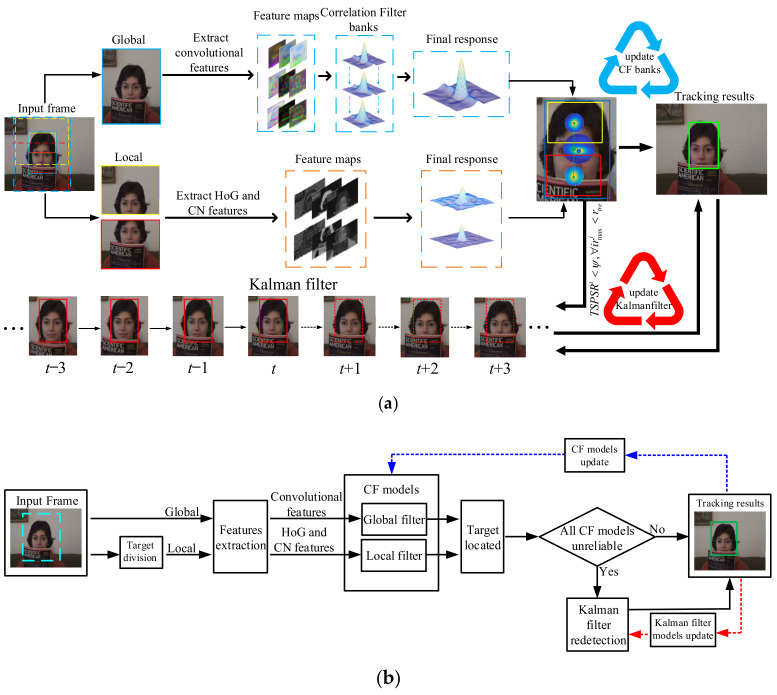
System overview of our proposed tracking algorithm. We divide the target according to its aspect ratio, and then use a combination of local filters and global filters to achieve accurate object tracking. When the robustness scores of the CF are lower than a certain threshold, we enable the KFR mechanism to recapture the target. (**a**) Visualization of data flow in our proposed tracking algorithm. (**b**) The functional components and their connections in our proposed tracking algorithm. The blue dashed line indicates the collaborative update strategy of the correlation filter (CF), and the red dashed line indicates that the Kalman filter (KF) needs to be updated after Kalman filter redetection (KFR) is turned on.

In this paper, we propose a novel local–global tracking algorithm to achieve the combination of multiple features and introduce a redetection mechanism based on Kalman filter, which can achieve accurate object tracking. The overall framework of our proposed algorithm is shown in Figure 2. Algorithm 1 describes the main steps of our proposed LGCF tracker.
**Algorithm 1** The Main Steps of Our Algorithm
**Input**: Initial object position
$p_{1}$ in the first frame, VGGNet-19 pretrained models;
**Output**:Estimated position
$p_{t}$; updated multiple correlation filters.

**Figure 3:**
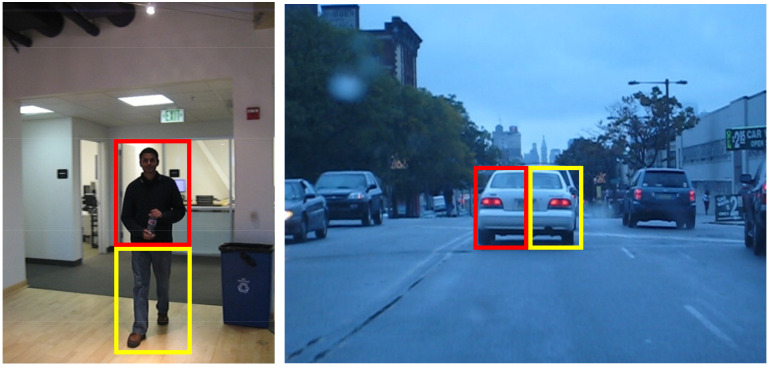
The way to divide the object when building two local filters. This layout is selected according to aspect ratio of the target object. Sequences are from Human2 and BlurCar1, respectively.

1. Divided target as Figure 3; 2. Initiate global filter models using Equation (19); 3. Initiate Kalman filter redetection models using Equations (22) and (23); 4. **for**
t = 2, 3, … **do** 5. Exploit the VGGNet-19 to obtain multi-layer deep features; 6. Compute the response maps of global filter using Equation (5); 7. Compute the TSPSR using Equations (28) and (29); 8. Find the final global target position $pos_{t}^{\left(g\right)}$ using Equation (17); 9. Compute the response maps of local filter using Equation (5); 10. Find the final local target position $pos_{t}^{\left(l\right)}$ using Equation (15); 11. **if**
$TSPSR^{t}<\psi ,\forall ir_{\max}^{i}<r_{tre}$
**then** 12.   Obtain the target position of the current frame by Kalman filter using Equation (26); 13.   Update Kalman filter redetection models using Equations (24)–(27); 14. **else** 15.   Obtain the target position of the current frame by multiple correlation filters using Equation (16); 16. **end if** 17. Update global filters using Equation (19); 18. Update local filters using Equation (20); 19. **end for**

First, our tracker consists of two parts: the global CF and the local CFs. The global CF is trained by multi-layer convolutional features extracted from VGGNet-19, while the local CF is trained by a combination of 31 dimensional HoG features and 11 dimensional CN features. We directly solve the ridge regression problem in the linear space to learn our global filter, but map the ridge regression to the non-linear space through the kernel function to obtain the local filter. Our global filter and local filter can work together to make full use of the global overall information and local detailed information of the target. At the same time, the global and local filters can achieve information transfer and mutual correction.

Secondly, based on our local–global model, we propose a local–global filter collaborative update strategy. According to the different states of the local filter, we divide the target tracking states into three cases: no occlusion, partial occlusion, and complete occlusion. An optimal update strategy for the global filter is given for different tracking situations. This update strategy can prevent our filter from learning the fake target’s appearance and error accumulation.

Finally, we propose TSPSR to measure the reliability of the current tracking results. Under normal circumstances, the tracking results calculated by the CF are fed to the KF for parameter update. In some challenging situations such as target loss, out-of-view rotation, or complete occlusion, the KF completely relies on the status and parameters of the target in the previous frame to predict the target position in the current frame. When the CF tracking results are unreliable, we will perform a resampling operation, and then use the KFR to estimate the current correct target position.

### 3.2. Global Filter

We use convolutional feature maps extracted from VGGNet-19 to build the global appearance of the target and train our global filter. $x_{l}\in R^{M\times N\times D}$ is denoted as the convolutional feature maps of size M×N×D from the *l*-th layer in VGGNet-19. The differences of M, N, and D at the *l*-th level are not considered and $x_{l}$ is regarded as the training sample. Circular samples $x_{m,n},(m,n)\in \{0,1,\ldots ,M-1\}\times \{0,1,\ldots N-1\}$ are generated by circular matrices of $x_{l}$. Each circular sample $x_{m,n}$ corresponds to a two-dimensional Gaussian label function:

(1) $$y(m,n)=e^{-\frac{{\left(m-M/2\right)}^{2}+{\left(n-N/2\right)}^{2}}{2\sigma^{2}}},$$

where M and N represent the width and height of the convolutional feature map, respectively; σ is the width of the kernel, and the correlation filters w have the same size as $x_{m,n}$. The goal is to find a function $f\left(z\right)=w^{T}z$ that minimizes the squared error between the sample $x_{m,n}$ and the regression target $y\left(m,n\right)$:

(2) $$w^{*}=\underset{w}{\arg\min}\sum_{m,n}\left\|w\cdot x_{m,n}-y\left(m,n\right)\right\|+\lambda {\left\|w\right\|}_{2}^{2},$$

where λ(λ>0) is the regularization factor. The inner product is induced by a linear kernel, e.g., $w\cdot x_{m,n}=\sum_{d=1}^{D}w_{m,n,d}^{T}x_{m,n,d}$. There are many channels of convolutional features; in order to simplify the calculation, we use a linear kernel in Hilbert space to derive $f\left(x_{m,n}\right)$:

(3) $$f\left(x_{m,n}\right)=w\cdot x_{m,n}=\sum_{d=1}^{D}w_{n,m,d}^{T}x_{m,n,d}.$$

Similar to the method in [46], using Fast Fourier transform (FFT) in each channel, the filter $W^{d}$ can be solved in the frequency domain. Each channel of the learned filter is represented in the frequency domain as follows:

(4) $$W^{d}=\frac{Y⊙{\bar{X}}^{d}}{\sum_{i=1}^{D}X^{i}⊙{\bar{X}}^{i}+\lambda },$$

where d∈{1,2,…,D}, Y=F(y), $X^{d}=F(x^{d})$, and F(y) means Discrete Fourier transform (DFT). The bars above the variables represent complex conjugates. The operator ⊙ represents a Hadamard (element-wise) product. In the next frame, the convolutional features of the Region of Interest (ROI) are extracted, where the *l*-th layer is defined as $z_{l}\in R^{M,N,D}$. The layer *l* response map $R_{l}$ is calculated as follows:

(5) $$R_{l}=F^{-1}(\sum_{d=1}^{D}W^{d}⊙{\bar{Z}}^{d}).$$

The operator $F{\left(\cdot \right)}^{-1}$ denote the inverse Fourier transform operation. We can obtain the estimated position of the target given by the *l*-th layer by searching the maximum value in the response map. We comprehensively consider the response maps generated by multiple feature layers. Specifically, we fuse the response maps of multiple convolutional layers by weights. The position of the maximum value of the weighted fusion response map is used to estimate the final position of global tracking:

(6) $$\hat{R}(m,n)=\alpha_{1}R_{1}(m,n)+\alpha_{2}R_{2}(m,n)+\ldots +\alpha_{l}R_{l}(m,n),$$

(7) $$(x^{\left(g\right)},y^{\left(g\right)})=\arg{}_{m,n}\max\hat{R}(m,n),$$

(8) $$pos^{\left(g\right)}=(x^{\left(g\right)},y^{\left(g\right)}),$$

where $\alpha_{l}$ is the weight of the *l*-th layer convolution feature response map. $pos^{\left(g\right)}$ is the target position estimated by our global filter.

### 3.3. Local Filter

Our local filter does not rely on local variability between different targets and uses the variant of HoG [47] features and color name (CN) [48] features to capture the gradient and color information of the target, respectively. HoG features and CN features have low dimensionality, so we refer to the method in KCF [8] and use the Gaussian kernel function to map the ridge regression to a nonlinear space to construct two local trackers. For different parts of the target, each tracker is considered as a separate tracker. At the same time, they have constraints on each other and there are also constraints between partial filters and global filters. We refer to the block method in [21]. According to the aspect ratio of the target, we use two methods of horizontal block and vertical block to split the target, as the Figure 3 shows.

Using the kernel function to extend the ridge regression (Equation (2)) to the non-linear domain, we can obtain the closed-form solution of the best filter kernel version:

(9) $$\alpha_{i}={\left(K+\lambda I\right)}^{-1}y_{i}.$$

Note that the subscript in Equation (9) indicates the *i*-th local filter. $\alpha_{i}$ represents the form of the filter $h_{i}$ in the dual domain. $K_{ij}=\kappa (x_{i},x_{j})$ is the kernel matrix, I is the identity matrix, and $y_{i}$ is a vector form of the desired output $y_{i}$.

For a kernel matrix with a cyclic structure, it is easy to construct a kernel solving Equation (2) without considering all the samples generated by the cyclic shifts. Given a kernel matrix $K=C(k^{xx})$ $k^{xx}$ is the kernel correlation of x with itself, where C(·) represents the cyclic data matrix generated by concatenating all possible cyclic shifts. The closed-form solution can be written as:

(10) $$\alpha_{i}=F^{-1}\left(\frac{F(y_{i})}{F(k_{i}^{xx})+\lambda }\right).$$

We use the HoG and CN features to construct our local target appearance representation and use the following Gaussian kernel to construct the kernel matrix:

(11) $$k^{xx^{\prime }}=\exp\left(-\frac{1}{\sigma^{2}}\left({\left\|x\right\|}^{2}+{\left\|x^{\prime }\right\|}^{2}-2F^{-1}\left(\sum_{c}{\hat{x}}_{c}^{*}⊙{{\hat{x}}^{\prime }}_{c}\right)\right)\right),$$

where $\hat{x}$ is the Fourier transform of x, ${\hat{x}}^{*}$ is the complex conjugate of $\hat{x}$, and ⊙ is element-wise. For the trained local filter $\alpha_{i}$, an image block of the same size as $x_{i}$ is cropped and its feature is z. Confidence maps of the local filter and predicted target position are calculated as follows:

(12) $${\hat{R}}_{i}=F^{-1}(F(k_{i}^{xz})⊙F(\alpha_{i})),$$

(13) $$(x_{i}{}^{\left(l\right)},y_{i}{}^{\left(l\right)})=\underset{m,n}{\arg\max}{\hat{R}}_{i}(m,n),$$

where $k_{i}^{xz}$ represents the kernel correlation of extracted feature $z_{i}$, and the learned partial appearance model $x_{i}$. ${\hat{R}}_{i}$ represents the response map of the *i*-th local filter. We use the midpoint of the predicted position of the two local filters as the final position $pos^{\left(l\right)}$ of local tracking:

(14) $$(x^{\left(l\right)},y^{\left(l\right)})=(\frac{x_{1}{}^{\left(l\right)}+x_{2}{}^{\left(l\right)}}{2},\frac{y_{1}{}^{\left(l\right)}+y_{2}{}^{\left(l\right)}}{2}),$$

(15) $$pos^{\left(l\right)}=(x^{\left(l\right)},y^{\left(l\right)}),$$

### 3.4. Local–Global Collaboration Model

We propose a global-to-local tracking algorithm, using the method in Figure 2 to build the target appearance model. Our global tracker is trained by convolutional features from the VGGNet-19 network, and the local tracker is trained by 31 dimensional HoG and 11 dimensional CN features. In the tracking process, the position and state of all filters are considered comprehensively to predict the final position of the target.

Specifically, whether the current local part of the target is occluded is determined by the maximum value of the local filter. When the maximum value of the local filter response $r_{\max}^{i}$ is less than the threshold $r_{tre}$, we consider that the current local tracker results are not reliable. Considering the relationship between the local prediction position and the global prediction position, we set a reliable circle area around the global prediction position, the radius of which is related to the target scale, and the radius value is $r=\sqrt{W^{2}+H^{2}}$. The Euclidean distance between the predicted position of the global tracking and the final position of the local tracking is calculated as $d=\left\|pos^{\left(g\right)}-pos^{\left(l\right)}\right\|$. To figure out whether the final position of the local tracking is within the reliable circle, that is, the relationship between r and d, the final tracking position is obtained as follows:

(16) $$pos=\left\{\begin{array}{ll} pos^{\left(g\right)} & \mathrm{if}\;d<\theta \cdot r\\ (1-\beta )pos^{\left(g\right)}+\beta pos^{\left(l\right)} & \mathrm{if}\;d>\theta \cdot r,\exists ir_{\max}^{i}>r_{tre}\\ \mathrm{KFR} & \mathrm{if}\;d>\theta \cdot r,\forall ir_{\max}^{i}<r_{tre} \end{array}\right.,$$

where $\Delta_{t-1}^{\left(l\right)}$ is the displacement vector of the center position of the two local correlation filters at time t relative to time t−1.

For the global filter, the filter of the first layer can be updated by minimizing the output error of all training samples, which still includes solving the correlation filters of D channels. Online learning will consume a lot of time, so we update the numerator $A_{t+1}^{d}$, and denominator $B_{t+1}^{d}$, of the global filter $H_{t+1}^{d}$, respectively, as follows:

(17) $$A_{t+1}^{d}=(1-\eta )A_{t}^{d}+\eta_{g}G⊙{\bar{F}}_{t}^{d},$$

(18) $$B_{t+1}^{d}=(1-\eta )B_{t}^{d}+\eta_{g}\sum_{i=1}^{D}F_{t}^{i}⊙{\bar{F}}_{t}^{i},$$

(19) $$H_{t+1}^{d}=\frac{A_{t}^{d}}{B_{t}^{d}+\lambda_{g}},$$

where $\eta_{g}$ is the update factor of the global filter. We use the maximum value of the local filter to determine whether the current target is occluded and whether the local–global learning model is updated is based on the state of the two local filters. We divide the tracking target status into the following three cases: no occlusion, partial occlusion, and complete occlusion. For three different cases, the global filter update method is as Table 1.

For local filters, we consider the state of each local filter separately. According to the maximum response values of the two local filters, the update method is selected as follows:

(20) $$\alpha_{i}^{t}=\left\{\begin{array}{ll} (1-\eta_{l})\alpha_{i}^{t-1} & if\;r_{\max}^{i}>r_{tre}\\ \alpha_{i}^{t-1} & otherwise \end{array}\right.,$$

where $\eta_{l}$ is the update factor of the local filter. In the next section, we will discuss our proposed TSPSR index and how we recapture targets using KF when the local–global multiple CFs tracking model fails to track the target.

### 3.5. Kalman Filter Redetection

Most CF-based trackers and KCF trackers only consider appearance change, fail to utilize the motion information of the target, and rely too much on the maximum response value to determine the target position. When the target is partially or completely occluded or is in situations such as fast motion and complex background, the tracker may lose the target. Therefore, we propose a redetection module based on KF for the local–global tracker. KF is an algorithm that makes an optimal estimation of the current true status based on the current measurement. The equation of the KF is divided into two parts: the time update equation and the measurement update equation. The time update equation uses historical predictions to estimate the current state, which can be regarded as a prediction equation; the measurement update equation adjusts the estimation of the target status through the current actual measurement, which can be regarded as a correction equation. The prediction equation can be expressed as:

(21) $${\hat{x}}_{k|k-1}=F_{k}{\hat{x}}_{k-1|k-1}+B_{k}u_{k},$$

where ${\hat{x}}_{k|k-1}$ represents the target state vector predicted at time k, $u_{k}$ is the control variant, and $B_{k}$ is the control matrix, which shows how the control variant $u_{k}$ acts on the current state. $\hat{x}$ is a four-dimensional vector $\left[\begin{array}{cccc} x & y & dx & dy \end{array}\right]$, where x and y represent the coordinates of the center position of the target, and dx, dy represent the speed of the target. $F_{k}$ is the state transition matrix, which is expressed as:

(22) $$F_{k}=\left[\begin{array}{cccc} 1 & 0 & 1 & 0\\ 0 & 1 & 0 & 1\\ 0 & 0 & 1 & 0\\ 0 & 0 & 0 & 1 \end{array}\right].$$

We set $P_{k|k-1}$ to the covariance matrix of the posterior estimation error at time k; the transfer process of the noise covariance matrix can be expressed as:

(23) $$P_{k|k-1}=F_{k}P_{k-1|k-1}F_{k}^{T}+Q_{k},$$

where the covariance matrix $Q_{k}$ represents the error caused by the prediction model at time k. In the updating equation, the KF uses the current measurement to correct the estimated results given by the prediction equation. The observed value $Z_{t}$ can be expressed as:

(24) $$Z_{k}=H_{k}{\hat{x}}_{k|k-1}+V_{k}.$$

The role of $H_{k}$ is to transform the state space to the measurement space, and $V_{k}$ is the observation noise at time k. We use $R_{k}$ to represent the error covariance matrix of the observational measurement $Z_{k}$. The role of Kalman coefficient $K_{k}$ is to weigh the magnitude of the predictive error covariance matrix $P_{k|k-1}$ and the observational measurement error covariance matrix $R_{k}$, determine which of the prediction model and the observation model is more reliable, and switch the system from the observation domain to the state domain. The Kalman coefficient $K_{k}$ can be expressed as:

(25) $$K_{k}=P_{k|k-1}H_{k}^{T}{\left(H_{k}P_{k|k-1}H_{k}^{T}+R_{k}\right)}^{-1}.$$

Based on what has been mentioned above, we update our KF using the following equation:

(26) $${\hat{x}}_{k|k}={\hat{x}}_{k|k-1}+K_{k}(z_{k}-H_{k}{\hat{x}}_{k|k-1}),$$

(27) $$P_{k|k-1}=(I-K_{k}H_{k})P_{k|k-1}.$$

**Figure 4:**
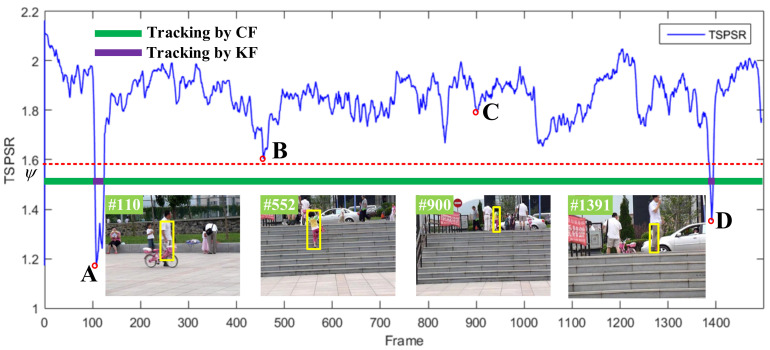
Distributions and analysis of time-space peak to sidelobe ratio (TSPSR) value on the Girl2 sequence. The green line represents the stage of tracking by CF, and the purple line represents the stage of tracking by KF. The four points A to D correspond to different frames, and the tracking model is selected according to the value of TSPSR.

When the local–global tracker is tracking normally, we input the results of the trackers as observations into the KF for parameter update. When target occlusion or tracking failure occurs, we completely use KF to predict the movement of the target. We use the Peak to Sidelobe Ratio (PSR) value of the global filter response map and the maximum value of the local filter response map to judge the reliability of the tracker. The calculation of PSR is as follows:

(28) $$PSR_{l}^{t}=\frac{\max(R_{l}^{t})-\mu_{l}^{t}}{\sigma_{l}^{t}},$$

where $R_{l}^{t}$ is the response graph of the global filter on the *l*-th layer at time t, $\max(R^{t})$ represents the maximum value of the global filter response map, and $\mu^{t}$ and $\sigma^{t}$ are the mean and standard deviation of the response map, respectively. When the target starts to enter the occlusion situation, the PSR value will decrease; when the target is completely occluded, the filter will learn the appearance characteristics of the occluded, and the PSR value will increase instead. In addition, different convolutional layers have different sensitivities to occlusion or rotation. The lower level convolutional features contain more spatial detail information and are more sensitive to changes in the appearance of the target. Therefore, we propose a time-space weighted PSR (TSPSR), which is calculated as follows:

(29) $$TSPSR^{t}=\sum_{t=1}^{T}\omega_{t}\frac{1}{L}\sum_{l=1}^{L}\beta_{l}PSR_{l}^{t},$$

where $\beta_{l}$ is the spatial weighting coefficient for the current *l*-th layer, and $\omega_{t}$ is the time weighting coefficient for t frames before the start of the current frame. In our algorithm, the switching mechanism for the correlation filter-based tracker (CFT) and Kalman filter based tracker (KFT) is as follows:

(30) $$\left\{\begin{array}{cc} TSPSR^{t}<\psi ,\forall ir_{\max}^{i}<r_{tre} & tracking\;by\;\mathrm{KFT}\\ otherwise & tracking\;by\;\mathrm{CFT} \end{array}\right.,$$

where ψ is the TSPSR threshold we set. TSPSR can effectively reflect the reliability of the current correlation filter tracking results. Figure 4 intuitively reflects the distribution of TSPSR on the Girl2 sequence. In this figure, we use green to represent the stage of tracking by CF, and purple to represent the stage of tracking by KF. Points B and C indicate that the girls in frame #552 and frame #900 are not occluded, and the TSPSR value is higher than the threshold; therefore, we use CF for tracking. However, the two points A and D indicate that the girl is occluded by a pedestrian in frames #110 and frame #1391, and the TSPSR value is lower than the threshold; therefore, we use KF for tracking.

## 4. Performance Analysis

In this section, we perform extensive experiments for the proposed algorithm using several benchmarks. First, we describe the detailed implementation and parameter settings of our tracker. Second, we analyze the effectiveness of each contribution in our proposed algorithm. Finally, we present the tracking performance of our proposed tracker compared with some state-of-the-art trackers. We perform qualitative-, quantitative-, and attributes-based evaluations on the OTB-2013 and OTB-2015 datasets to evaluate the performance of our algorithm.

### 4.1. Experimental Setup

#### 4.1.1. Implementation Details

Our proposed tracker was implemented in MATLAB 2016b on a computer with an Intel (R) Xeon (R) CPU e5-2640 2.40 GHz CPU and a NVIDIA GeForce GTX1080Ti GPU (11 g memory) (OMNISKY, Beijing, China). The CUDA version is 10.1. We use MatConvNet which is a common deep learning toolbox in MATLAB to implement the extraction of convolutional features of VGGNet-19. More specifically, parameters of the tracker are set as follows: the kernel width of the local filter σ is set to 0.1, the global regularization parameter $\lambda_{g}$ is set to 0.0001, and the local learning rate $\eta_{l}$ is set to 0.18. We use the features of conv3-4, conv4-4, and conv5-4 of VGGNet-19 with their weights set to 0.25, 0.5, and 1, respectively. The local tracker reliability threshold $r_{tre}$ is set to 0.55, In the TSPSR, the first five frames are taken, the spatial weight vector $\beta_{l}$ is set to [0.25 0.5 0.1], the time weights vector of the previous five frames $\omega_{t}$ are set to [0.4 0.2 0.2 0.1 0.1], and the confidence circle radius weight is set to 0.9.

#### 4.1.2. Datasets

In order to fully verify the effectiveness of our proposed algorithm, we perform comparative experiments on the OTB-2013 and OTB-2015 datasets, which contain 50 image sequences and 100 image sequences, respectively. These two datasets contain 11 common challenge attributes, including illumination variation (IV), occlusion (OCC), scale change (SV), fast motion (FM), deformation (DEF), motion blur (MB), out-of-view (OV), background clutter (BC), low resolution (LR), in-plane rotation (IPR), and out-of-plane rotation (OPR).

#### 4.1.3. Evaluation Indicators

The One-Pass Evaluation (OPE) proposed in OTB-2013 [25] is used to objectively evaluate the performance of the tracker, mainly using two indicators: precision and success rate. Precision is defined as the percentage of frames whose average Euclidean distance between the center position of the tracked target and ground-truth is less than the given threshold. The success rate represents the percentage of successful frames whose overlap rate between the tracked bounding boxes and ground-truth is greater than the given threshold. The compared trackers are ranked by the area under the curve (AUC).

### 4.2. Effectiveness Analysis

#### 4.2.1. Analysis of Local–Global Multiple CF Learning Model

We design experiments to verify the effectiveness of our LGCF learning model, which are shown in Table 2. Global (baseline) represents that we use the global model as a baseline. Compared with a single global correlation filter model, it can be found that the local–global multiple correlative filter collaborative model achieves a 1.34% rise in accuracy and a 1.89% rise in success rate on OTB-2013, a 1.30% improvement in accuracy, and a 1.82% improvement in success rate on OTB-2015.

#### 4.2.2. Analysis of Collaborative Update Strategy

**Figure 5:**
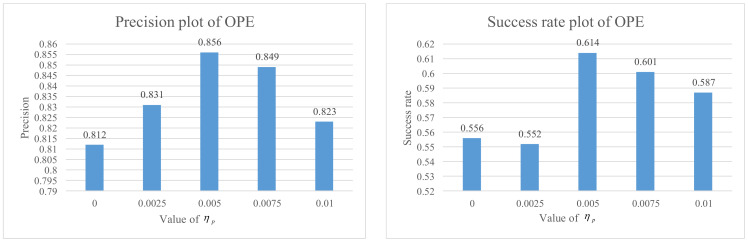
The experimental results for different reliability ratio threshold $\eta_{P}$ on OTB-2015.

As is shown in Figure 5, experimental results show the effectiveness of a local–global collaborative updating strategy on OTB-2013 and OTB-2015. We conduct experiments on the different values of the updating parameter $\eta_{P}$ in the partial occlusion of the global tracker. $\eta_{P}$ set to 0 represents whether the target is partially or fully occluded and the global tracker will stop updating. $\eta_{P}$ set to 0.01 represents only when the target is fully occluded and then the global tracker will stop updating. When the $\eta_{P}$ is set to 0.005, the best performance is obtained.

### 4.3. Overall Performance

We compare the proposed trackers with the other 12 state-of-the-art trackers, and we divided these trackers into the following categories: (1) DCF-based trackers: SRDCF [6], SAMF_AT [49], MUSter [50], DSST [33], LCT [22], Staple [51]. (2) CNN-based trackers: HCF [17], SiamFC [35], HDT [15], CFNet [52], DeepSRDCF [13]. (3) Set-based tracker: MEEM [53].

#### 4.3.1. Quantitative Evaluation

**Figure 6:**
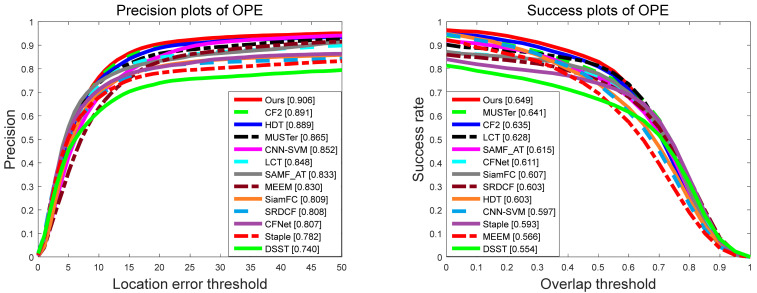
Precision and success rate of our proposed tracker and compared other 12 trackers on OTB-2013.

**Figure 7:**
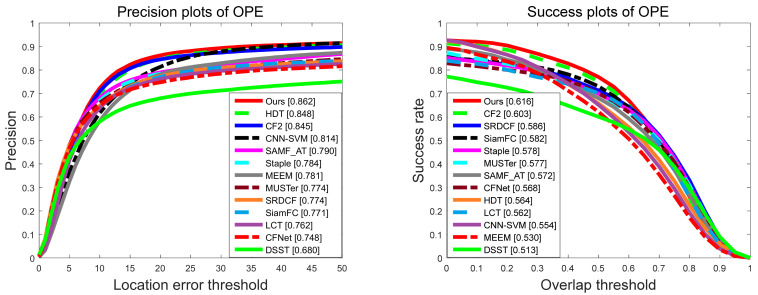
Precision and success rate of our proposed tracker and compared other 12 trackers on OTB-2015.

We compare the overall accuracy and success rate of the proposed tracker with the other 12 trackers on OTB-2013 and OTB-2015. Figure 6 and Figure 7 show the evaluation results on these two datasets. It can be seen that compared with the other latest methods, our proposed tracker shows excellent performance in both success rate and accuracy, because the OTB-2015 dataset contains the OTB-2013 dataset but includes some more challenging sequences.

#### 4.3.2. Attribute-Based Evaluation

**Figure 8:**
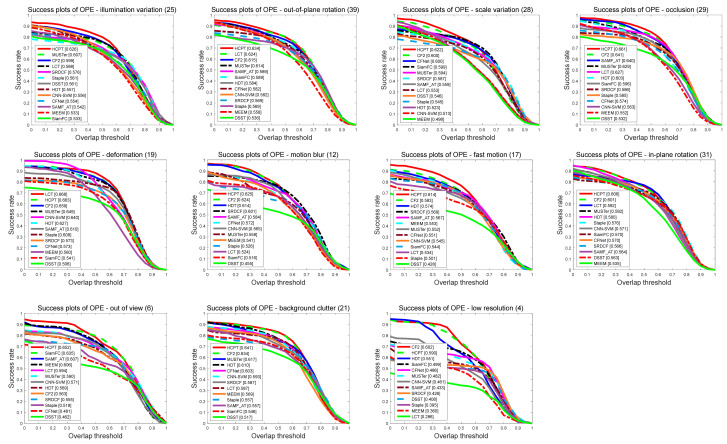
Success rate plots of 11 tracking challenging on OTB-2013. The plots illustrate the experimental results using 11 attributes. The legend shows the success rate and we show the results of 12 trackers.

**Figure 9:**
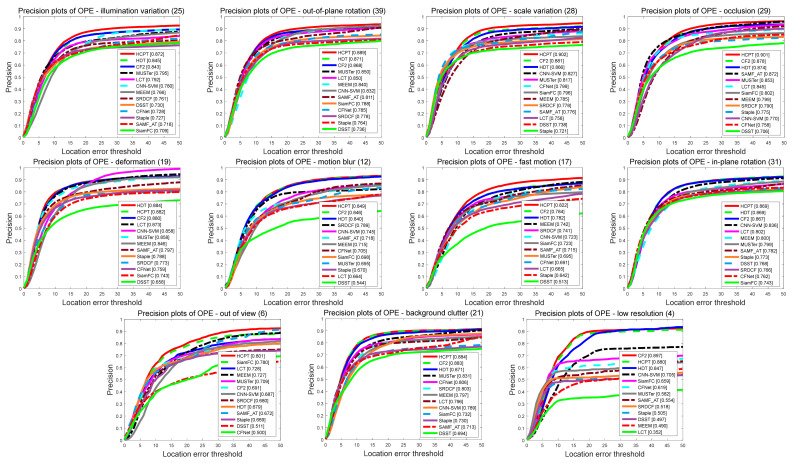
Precision plots of 11 tracking challenging on OTB-2013. The plots illustrate the experimental results using 11 attributes. The legend shows the success rate and we show the results of 12 trackers.

We use 11 challenging attributes on OTB-2013 and OTB-2015 to comprehensively evaluate the accuracy and robustness of our proposed tracker in various challenging scenarios. Figure 8 and Figure 9 show the evaluation results of our tracker and the other 12 state-of-the-art trackers based on different attributes on OTB-2013, respectively. Table 3 shows the results of the attribute-based accuracy rate on OTB-2015. It can be seen from the figure that our proposed algorithm performs well in most of the 11 attributes, especially in the 2 attributes OV and OCC. Owing to our local tracking module and KFR module, it can effectively deal with challenging situations such as out-of-view and occlusion. In addition, the local–global collaborative updating strategy enables our filter to correctly learn the appearance of the tracking target, and avoids learning the appearance of foreground occlusions or backgrounds.

#### 4.3.3. Qualitative Evaluation

**Figure 10:**
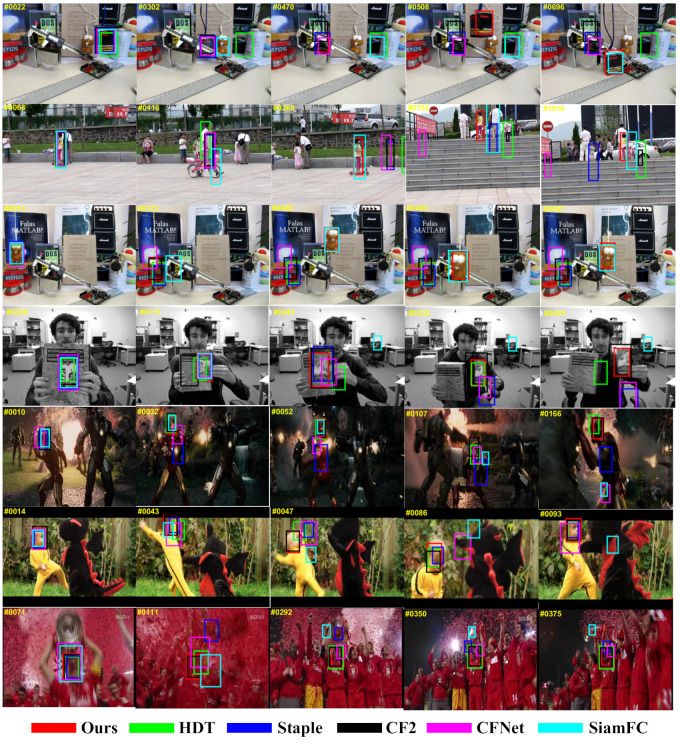
The comparisons of bounding box for different algorithms on 7 challenging image sequences of OTB-2013 and OTB-2015 (from top to down are Box, Girl2, Lemming, ClifBar, Ironman, DragonBaby, and Soccer, respectively).

Figure 10 shows the tracking results of our proposed tracker and five other trackers (including Staple [51], HDT [15], CFNet [52], SiamFC [35], and HCF [17]) on ten challenging video sequences. These frame sequences are from the public benchmarks OTB-2013 [25] and OTB-2015 [26]. In general, our tracker can track targets more accurately. In the “Box” sequence, the target is almost completely occluded at the 47th frame, and our tracker can still track it accurately. At the 508th frame, only our tracker can track the object correctly. In the “Girl2” sequence, when the little girl is completely occluded by a pedestrian and then appears in view, only our tracker can accurately recognize it, which contributes to our KFR mechanism. For “Lemming” sequences with OCC, IPR, and IR challenges, occlusion at the 372nd frame forces most trackers to lose their targets. In the 408th frame, only our tracker and SiamFC could accurately track the target. For “Soccer”, “Skating1”, and “Ironman” sequences with IV and BC, our tracker copes with illumination variation and background clutter very well. For “Dragonbaby” sequences with OPRs and OV challenges, SiamFC and CFNet have lost targets, but our tracker is still able to accurately capture targets. At the same time, as shown in the “Trellis 1” sequence with the SV challenge, our tracker can also handle scale changes very well. For “Clifbar” and “MotorRolling” sequences with BC, IV, and FB challenges, the other five methods perform poorly, but our tracker can handle this complex situation.

## 5. Conclusions

This paper proposes a local–global multiple correlation filters-based object tracking algorithm. First of all, global filters and local filters transferring information to each other can effectively cope with the challenges of occlusion and rotation with the comprehensive use of convolutional features and hand-crafted features. Based on our learning model, we propose a local–global collaborative updating strategy, which can prevent the tracker from learning the appearance of foreground occlusions or background clutter. In addition, we propose the time-space peak to sidelobe ratio (TSPSR) index, which can effectively reflect the reliability of the current KF tracker. When the current KF fails, the KFR model can be used to accurately recapture the target and predict the position of the original target. The experimental results on the OBT-2013 and OTB-2015 show that our tracker performs favorably against the other latest 12 trackers over accuracy and robustness.

## Figures and Tables

**Table 1:** Three different update states.

No	State of Tracker	State of Learning Rate	Value of Learning Rate	Condition in *t*-th Frame
1	No occlusion	Normal update	$\eta_{n}$	$\forall ir_{\max}^{i}>r_{tre}$
2	Partial occlusion	Slow update	$\eta_{p}$	$\exists ir_{\max}^{i}>r_{tre}$
3	Complete occlusion	Not update	$\eta_{c}$	$\forall ir_{\max}^{i}<r_{tre}$

**Table 2:** Effectiveness study of proposed local–global cooperation mechanism and KFR.

Types of Models	OTB-2013 Precision	OTB-2013 Success Rate	OTB-2015 Precision	OTB-2015 Success Rate
Global (Baseline)	0.891	0.635	0.845	0.603
Global + Local	0.903	0.647	0.856	0.614
Global + Local + KFR	0.906	0.649	0.862	0.616

**Table 3:** Performance evaluations of different attributes on OTB-2015. We use red and blue fonts to mark the top two scores.

Trackers	IV	OR	SV	OCC	DEF	MB	FM	IR	OV	BC	LR
CNN-SVM	0.789	0.806	0.791	0.73	0.793	0.767	0.742	0.798	0.659	0.776	0.79
SRDCF	0.786	0.724	0.75	0.703	0.699	0.782	0.762	0.739	0.572	0.775	0.631
Staple	0.776	0.738	0.739	0.728	0.751	0.719	0.709	0.772	0.644	0.749	0.591
MUSTer	0.776	0.753	0.72	0.734	0.689	0.699	0.684	0.756	0.603	0.784	0.677
LCT	0.739	0.748	0.687	0.682	0.689	0.673	0.667	0.768	0.616	0.734	0.49
MEEM	0.733	0.795	0.74	0.741	0.754	0.722	0.728	0.79	0.690	0.746	0.605
SiamFC	0.728	0.75	0.739	0.722	0.69	0.724	0.741	0.752	0.65	0.69	0.815
SAMF_AT	0.723	0.764	0.75	0.748	0.687	0.765	0.718	0.778	0.652	0.713	0.716
DSST	0.714	0.641	0.655	0.597	0.542	0.595	0.562	0.689	0.483	0.704	0.55
CFNet	0.686	0.728	0.715	0.674	0.643	0.687	0.695	0.759	0.572	0.724	0.787
HDT	0.815	0.807	0.812	0.774	0.821	0.794	0.802	0.834	0.687	0.844	0.766
HCF	0.835	0.817	0.807	0.776	0.791	0.804	0.792	0.849	0.689	0.852	0.822
Ours	0.87	0.85	0.835	0.817	0.806	0.846	0.796	0.856	0.784	0.851	0.867

* For the scores in Table 3, 11 columns represent the performance of different algorithms under 11 challenge attributes, and 13 rows represent the performance of 13 algorithms under different challenge attributes. The best and the second-best precision in each column are highlighted in red and blue respectively.

## Data Availability

Publicly available datasets were analyzed in this study. This data can be found here: http://cvlab.hanyang.ac.kr/tracker_benchmark/datasets.html.
